# Interaction between Cu and Thiols of Biological and Environmental Importance: Case Study Using Combined Spectrophotometric/Bathocuproine Sulfonate Disodium Salt Hydrate (BCS) Assay

**DOI:** 10.3390/molecules28135065

**Published:** 2023-06-28

**Authors:** Dora Crmarić, Elvira Bura-Nakić

**Affiliations:** Division for Marine and Environmental Research, Ruđer Bošković Institute, Bijenička Cesta 54, 10 000 Zagreb, Croatia; dcrmaric@irb.hr

**Keywords:** copper, thiols, reduction, oxidation, kinetics

## Abstract

Considering the biological and ecological importance of Cu–thiol interactions and the discrepancies in previous research, this study focuses on Cu interactions with biologically and ecologically relevant thiols: glutathione (GSH), L-cysteine (L-cys), 3-mercaptopropionic acid (MPA), and thioacetic acid (TAA) in aqueous solution. The addition of Cu(II) to a thiol-containing solution led to a rapid reduction of Cu(II) and the formation of a Cu(I)–thiol complex. The mechanism of Cu(II) reduction and Cu(I) complex formation as well as the kinetics of Cu(I) oxidation strongly depend on the structural properties of the individual thiols investigated. The reducing power of the investigated thiols can be summarized as follows: L-cys ≅ GSH > MPA > TAA. The reaction order, with respect to Cu(I) oxidation, also changes over the time of the reaction course. The deviation of the reaction kinetics from the first order with respect to Cu(I) in the later stages of the reaction course can be attributed to a Fenton-like reaction occurring under low thiol concentration conditions. At high Cu:thiol ratios, in the case of GSH, L-cys, and MPA, the early stage of the reaction course is characterized by high Cu(I) stability, most likely as a result of Cu(I) complexation by the thiols present in excess in the reaction mixture.

## 1. Introduction

As one of the most abundant trace elements in living organisms, copper (Cu) is an essential metal in the biochemistry and physiology of all organisms [1]. Because of its ability to undergo redox changes between the oxidation states of Cu(I) and Cu(II), Cu is an important catalytic cofactor in redox reactions involved in electron transfer [2,3]. Thiol compounds are known to interact strongly with Cu and can inhibit Cu-containing lysyl oxidase and tyrosinase enzymes [4,5,6,7]. Furthermore, biological thiols containing Cu in the active sites are an example of substrates used by copper–zinc superoxide dismutase (Cu/Zn-SOD), which catalyzes the dismutation of superoxide radicals to oxygen and hydrogen peroxide [8,9]. However, Cu(II) reduced to Cu(I) by hydrogen peroxide, superoxide, or thiol compounds can catalyze the formation of reactive oxygen species (ROS) that can lead to cell damage, with Cu acting as a pro-oxidant in this scenario [10,11]. Winterbourn et al. reported that thiols, especially aminothiols, such as cysteine, enhance the reduction of the Cu active site of CuZn-SOD and lead to the formation of hydrogen peroxide and disulfide, though CuZn-SOD is less reactive than free Cu(II) [12]. Many authors attribute Cu toxicity to the oxidative damage caused by free radicals, which leads to DNA damage, increased lipid peroxidation, or decreased enzymatic activity [13,14,15,16]. Murakami et al. showed that Cu ions inhibit GSH reductase activity, with Cu(I) ions acting more strongly than Cu(II) ions, which is consistent with previous studies by Xiao et al. [17,18]. Considering Cu toxicity, it is crucial to maintain Cu homeostasis in cells. To this end, cells have evolved a sophisticated machinery that includes multiple Cu ion transporters and chaperones that transport Cu to proteins [19]. Thiols are widely distributed in Cu-rich proteins and Cu chaperones. In eukaryotic organisms, Cu transporter 1 (CTR1) is responsible for binding Cu(II) via the N-domain of CTR1, reducing Cu(II) ions to Cu(I) and transferring Cu(I) ions to the cytoplasmic domain—a complex process involving the amino acids methionine, histidine, and cysteine in Cu binding [20,21]. Once Cu has entered cells, it binds to Cu chaperones, e.g., antioxidant 1 (ATOX1), which is the Cu chaperone for super-oxide dismutase (CCS), and the cytochrome C oxidase Cu chaperone (COX17), whose homologues in yeast are found in bacteria and thiol molecules [22]. Hatori et al. found that the cellular glutathione/glutathione disulfide pair (GSH/GSSG) prevents the oxidation of cysteines of ATOX1 and thereby regulates Cu binding to ATOX1 [23]. Furthermore, the importance of Cu–thiol interactions has been demonstrated in yeast CuCOX17, where Cu atoms are trigonally coordinated with thiolates, and in human COX17, where cysteine and glutathione are involved in Cu(I) binding and transfer to the cochaperones SCO1 and COX1 [24,25].

In addition, thiol compounds may play an important role in binding Cu in marine waters and detoxifying Cu in marine phytoplankton. It is known that marine cyanobacteria produce strong Cu-binding ligands in response to Cu pollution. Consequently, biologically produced ligands may contribute significantly to the pool of strong binding ligands classified as L1 [26,27]. Although the exact composition of Cu-binding ligands in seawater is not yet known, Whitby et al. suggested that thiourea (TU)- and glutathione (GSH)-like thiols contribute significantly to the Cu-binding ligand pool, resulting in strong Cu complexation and, consequently, femtomolar concentrations of free toxic Cu [28]. The authors also suggested that other thiols, such as cysteine, 3-mercaptopropionic acid, and 2-mercaptoethanol, may play an important role in Cu binding in marine waters. Tang et al. reported an increased GSH release in the diatom *Thalassiosira weissflogii* after exposure to elevated inorganic Cu concentrations [29]. However, they suggested that this was due to cell membrane damage caused by the increased Cu rather than a cellular mechanism that counteracts Cu toxicity [29]. However, they suggested that GSH release by diatoms may contribute significantly to the Cu-binding ligand pool in marine waters [29]. Because of the biological and ecological importance of Cu–thiol complexes, Cu interactions with thiols have been of interest for many years, though there are discrepancies in the understanding of the exact reaction mechanisms and Cu oxidation state in such reactions [30]. Nevertheless, Smith et al. reported that the stabilization of the free radical intermediate in Cu-catalyzed thiol oxidation varies as a result of structural differences between thiols affecting the oxidation rate, which is further discussed in their paper [31]. Considering the biological and ecological importance of Cu–thiol interactions, as well as the discrepancies in the reaction mechanisms proposed so far, this study focuses on Cu interactions with biologically relevant thiols: glutathione (GSH), L-cysteine (L-cys), 3-mercaptopropionic acid (MPA), and thioacetic acid (TAA).

The tripeptide GSH contains the amino acids glycine, cysteine, and glutamic acid, with the cysteine component providing the reactive thiol group. GSH has been found in many prokaryotic and most mammalian cells in a concentration range of 0.2–10 mM [32]. Since GSH is present in at least a twofold excess over Cu, it plays an important role in cellular Cu uptake, where Cu binds to GSH as a Cu(I) complex shortly after entering the cell and is transferred in complexed form to metallothionein [33,34]. The Cu-buffering role of GSH was demonstrated in *Streptococcus pyogenes*, where only GSH that was strongly protected against Cu toxicity and excess Cu showed no toxic effects when GSH was abundant [33]. Endogenous GSH also plays an important role in the antioxidant defense of plants against abiotic stress by scavenging ROS [35]. In aquatic environments, processes such as cell lysis and exudation can lead to the release of low molecular weight (LMW) thiols, including GSH, into natural waters [36,37]. Although GSH is abundant in micromolar concentrations in many organisms, GSH concentrations in natural waters are usually in the nanomolar range [38,39,40]. Increasing the concentration of Cu in water promotes the release of GSH, but the exact form of GSH in water and the mechanisms of Cu–GSH complex formation are still under investigation [39]. It has been suggested that GSH measured in water may be in its oxidized form, i.e., glutathione disulfide (GSSG), or in the form of complexes with trace metals [39]. In addition to GSH, other LMW thiols, such as cysteine, have also been detected in lakes, estuaries, and marine waters and have been proposed as Cu(I)-binding ligands, though the exact mechanisms of Cu(I) complexation with thiols are still under investigation [41,42]. L-cysteine is a non-essential amino acid that plays an important role in protein synthesis and stability through the formation of disulfide bonds [43]. The reactivity of L-cys is due to the sulfhydryl group (-SH), and L-cys serves as a ligand for the binding of trace metals, including Cu, and for active sites in enzymes. Deprotonation of the sulfhydryl group produces a negatively charged and reactive thiolate anion that can participate in oxidation or alkylation by electrophiles [44]. The thiolate anion is known to form mononuclear complexes with Cu(I), including mononuclear mixed thiolate-nitrogen-bonded species as well as Cu(I)–thiolate clusters [45]. In addition to glutathione and L-cysteine, 3-mercaptopropionic acid (MPA) is widely distributed in freshwater and marine environments, where it may be of biological and abiotic origins [38]. Biologically produced MPA has been addressed as a metabolite product of other thiol species, but it is also found in the methanogens *M. Jannaschii*, where its presence is associated with the coenzyme M rather than the metabolism of other thiols [46,47]. Abiotically generated MPA may originate from the abiotic transformation of sulfur species, such as hydrogen sulfide (H_2_S) [46,47]. Although interaction with Cu ions has been reported in marine and freshwater systems, it has not been thoroughly investigated because of the rapid oxidation of MPA in natural environments and the presence of other possible MPA-binding trace metals, such as iron, Fe [38]. Finally, thioacetic acid (TAA) is a thiocarboxylic acid that has been implicated in the origin of life as a potential acetyl-CoA analogue in prebiotic chemistry [48,49,50]. The oxidation of TAA and ethanethiol (ET) by Fe(III) has already been studied, and the mechanism by which the Fe(III) is reduced to Fe(II) while oxidizing thioacetate to diacetyl disulfide, leading to the formation of thioester and iron sulfide (FeS), has been proposed [50]. However, similar interactions of Cu with TAA are not known so far.

In this manuscript we present a kinetic study of Cu(II) reduction with thiols of biological and ecological importance (GSH, L-cys, MPA, and TAA). The kinetics of Cu(II) reduction and the Cu(I) oxidation formed are evaluated under different experimental conditions, i.e., different Cu and thiol concentration ratios. Thiols such as GSH are present in excess in many prokaryotic and mammalian cells, as well as in the aquatic environment; therefore, we decided to investigate Cu(II) reduction and Cu(I) oxidation with excess thiol in relation to Cu [32,33,34,38,39,40]. However, previous research on the interactions of Cu with thiols, including GSH, lacks data on Cu(II) reduction when Cu(II) and GSH are present in equimolar ratios. Therefore, we decided to additionally investigate Cu(II) reduction in the presence of thiols when Cu:thiol = 1:1.

## 2. Results and Discussion

### 2.1. Interaction of Cu and GSH

**Figure 1:**
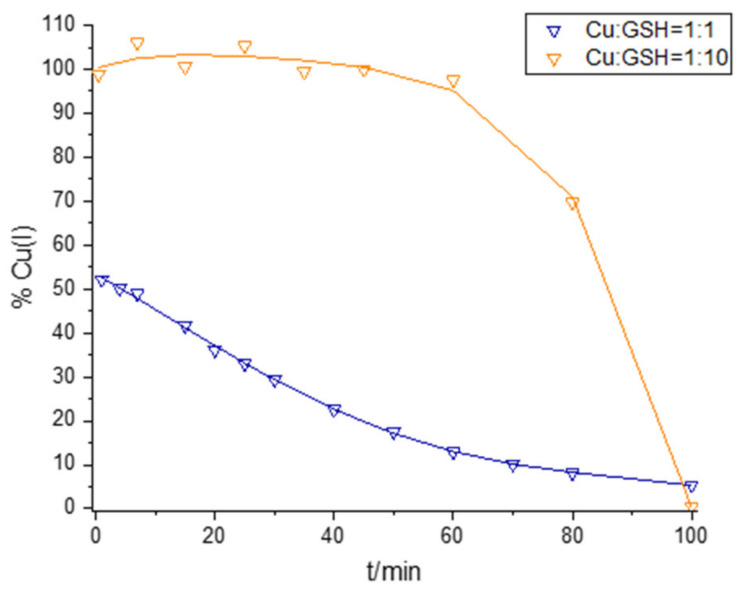
Kinetics of Cu(II) reduction and Cu(I) oxidation in solution containing 100 µM Cu(II) and either 100 µM GSH (blue line) or 1000 µM GSH (orange line).

**Figure 2:**
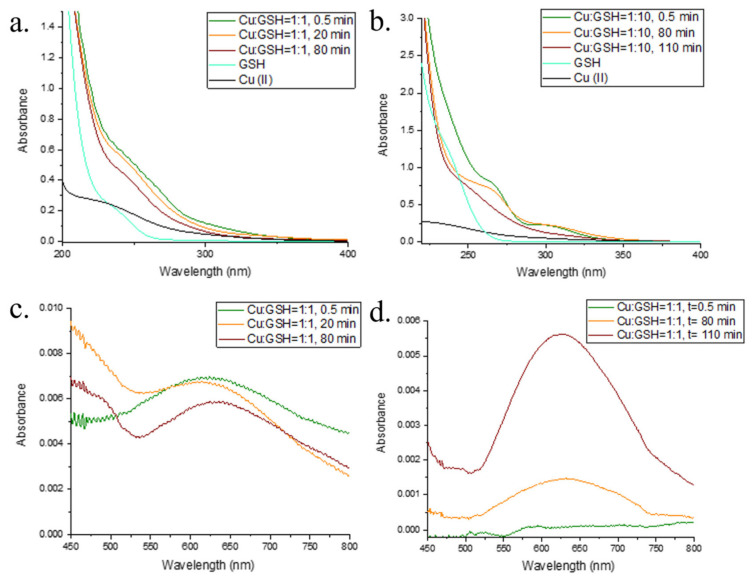
UV–Vis spectra of (**a**) Cu:GSH = 1:1 at t = 0.5 min (green line), t = 20 min (orange line), and t = 80 min (red line). UV–Vis spectra of a solution containing 100 µM GSH without Cu (cyan line) and 100 µM Cu without GSH (black line) are also shown. (**b**) Cu:GSH = 1:10 at t = 0.5 min (green line), t = 80 min (orange line), and t = 110 min (red line). The UV–Vis spectra of the solution with 1000 µM GSH without Cu (cyan line) and 100 µM Cu without GSH (black line) are also shown. (**c**) and (**d**) are as in (**a**) and (**b**), respectively, with the UV–Vis output wavelength changed to show changes during the course of the reaction at wavelengths above 450 nm.

The interactions of Cu(II) with GSH were studied at Cu:GSH ratios of 1:1 and 1:10 over the course of 100 min at pH = 8.4 in 0.1 M borate buffer under oxic conditions (Figure 1 and Figure 2). For both Cu:GSH ratios studied, Cu(II) reduction occurred immediately after the addition of Cu(II) to the buffered GSH solution, and Cu(I) concentrations were monitored using a BCS assay as described in Section 3.2. The kinetics of Cu(II) reduction and Cu(I) oxidation are shown in Figure 1. At a ratio of Cu:GSH = 1:10, all the Cu present in the solution was in its reduced form at the beginning of the reaction (the added Cu(II) was equal to Cu(I) detected using the BCS assay), implying the rapid reduction of Cu(II) in the presence of excess GSH. Oxidation of Cu(I) under the conditions of a tenfold excess of GSH with respect to the initial Cu(II) started after 60 min, and after 100 min, Cu(I) was no longer present in the solution. The UV–Vis spectra of Cu:GSH = 1:10 show peaks indicative of the ligand charge transfer band of a Cu(I)–GSH complex, with A_max_ values observed at 255 nm and 300 nm [51,52]. After 60 min, the oxidation of Cu(I) began and a decrease in A_255_ and A_300_ was also observed, while a peak characteristic of the Cu(II) complex with oxidized glutathione, Cu(II)–GSSG, appeared with an A_max_ of 625 nm [52,53]. A peak with an A_max_ of 250 nm was also observed in the solution with Cu:GSH = 1:1, but a peak at 300 nm was not present. A peak with an A_max_ of 625 nm, which can be attributed to the Cu(II)–GSSG complex, was present from the beginning of the reaction, which is consistent with Cu(I) accounting for a maximum of 52% of the Cu present in the solution. An examination of reaction kinetics (ln Cu(I) and 1/Cu(I) concentration–time) shows significant differences between the reaction kinetics of mixtures with Cu:GSH = 1:1 and those of mixtures with Cu:GSH = 1:10 (Figure S1 in the Supplementary Materials). In the mixture with Cu:GSH = 1:1, linearity with time can be observed in the ln Cu(I) concentration over the whole course of the reaction (100 min), implying first-order kinetics, with respect to Cu(I), with the reaction rate constant, *k*, reaching 0.0024 min^−1^. The plots of ln Cu(I) and 1/Cu(I) concentration–time in the case of a mixture with Cu:GSH = 1:10 in the first 60 min of the reaction course show reaction kinetics independent of the Cu(I) concentration. The later phase of the reaction is characterized by rapid Cu(I) oxidation. The rapid Cu(I) oxidation is enhanced by the formation of the Cu(II)–GSSG complex. The formation of the Cu(II)–GSSG complex inhibits the Fenton-like reversal reaction, as explained in the case of L-cysteine (see below) and in Kachur et al. and Moffet and Zika [54,55]. The early stage of the reaction, in the case of GSH excess, can be explained by strong interactions between the Cu(I) and GSH present in excess, which prevent fast Cu(I) oxidation with molecular O_2_. Ngamchuea et al. proposed a two-phase mechanism in which a Cu(II)–GSH complex with a stoichiometry of 1:2 is formed in the first phase, and the second phase comprises the Cu-catalyzed oxidation of GSH to GSSG [56]. In general, our results are in agreement with the two-phase mechanism of GSH oxidation proposed by Ngamchuea et al. [56]. However, while Ngamchuea et al. proposed the formation of a Cu(II) complex with GSH as an intermediate species in the first reaction phase, our results support the formation of a Cu(II) complex with oxidized GSH in the early as well as later stages of the reaction, depending on the Cu(II)-to-GSH ratio [57,58]. In the solution containing Cu:GSH = 1:1, a peak with an A_max_ of 625 nm was present from the very beginning of the reaction, indicating the presence of a Cu(II)–GSSG complex even at the early reaction stage. In contrast, for Cu:GSH = 1:10, a peak with an A_max_ of 625 nm was not present in the first reaction stage, since 100% of the Cu present in the solution was in its reduced form in the first reaction stage. For Cu:GSH = 1:10, a peak with an A_max_ of 625 was formed when Cu(I) oxidation began.

### 2.2. Interaction of Cu and L-cys

**Figure 3:**
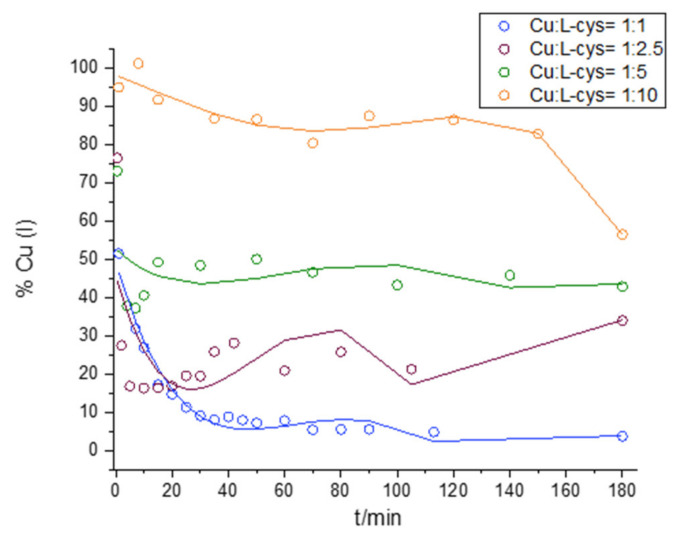
Kinetics of Cu(II) reduction and Cu(I) oxidation in solution containing 100 µM Cu(II) and 100 µM L-cys (blue line), 250 µM L-cys (wine red line), 500 µM L-cys (green line), or 1000 µM L-cys (orange line).

**Figure 4:**
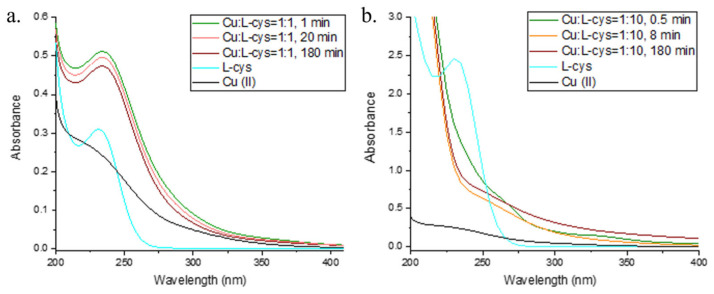
UV–Vis spectra of (**a**) Cu:L-cys = 1:1 at t = 1 min (green line), t = 20 min (orange line), and t = 180 min (red line). The UV–Vis spectra of a solution containing 100 µM L-cys without Cu (cyan line) and 100 µM Cu without L-cys (black line) are also shown. (**b**) Cu:L-cys = 1:10 at t = 0.5 min (green line), t = 8 min (orange line), and t = 110 min (red line). The UV–Vis spectra of the solution containing 1000 µM L-cys without Cu (cyan line) and 100 µM Cu without L-cys (black line) are also shown.

The kinetics of Cu(II) reduction and Cu(I) oxidation over time at Cu:L-cys ratios of 1:1, 1:2.5, 1:5, and 1:10 are shown in Figure 3 and Figure 4. Immediately after the addition of Cu(II) to the L-cys solution, the reduction of Cu(II) to Cu(I) was observed, as in the study of the interaction of Cu and GSH presented in this manuscript (Section 2.1.). The presence of Cu(I) was detected in all Cu:L-cys ratios studied during the 180 min, indicating the formation of a Cu(I) complex with L-cys that was resistant to oxidation with molecular O_2_. The amount of reduced Cu(II) and the stability of Cu(I) to oxidation increased with increasing Cu:L-cys ratios, as shown in Figure 3. The UV–Vis spectrum of L-cys alone in 0.1 M borate buffer is characterized by a peak with an A_max_ around 235 nm, which is consistent with the literature data [59]. Furthermore, Battin et al. reported A_max_ values at the same wavelength for all sulfur-mediated compounds, which prevented Cu-mediated DNA damage [51]. Immediately after the addition of Cu(II) to a solution containing L-cys and borate buffer, the previously observed peak at 230 nm disappeared, indicating the loss of free L-cys. At the same time, two new peaks appeared with A_max_ values of 260 nm and 336 nm. Similar peaks were also observed in studies by Pecci et al. and Rigo et al. and were attributed to Cu(I)–cys complexes, with a difference in the position of the second peak (300 nm), which could be due to the different reaction conditions (different buffers and pH values, and different oxic and anoxic reaction conditions) [60,61]. Cavalini et al. reported a compound with an A_max_ of 330 nm at a pH of >8 as an intermediate Cu(II)–cysteine complex with a stoichiometry of 1:2, which disappeared after cysteine oxidation during the reaction [59]. Also in our case, the peaks at 260 and 330 nm disappeared during the course of the reaction when L-cys was oxidized and cystine was formed, which was confirmed by an increase in A in the 200–400 nm region where cystine absorbs [61].

An analysis of reaction kinetics (ln Cu(I) and 1/Cu(I) concentration–time plots) in all four model solutions containing a fixed Cu(II) concentration and in which L-cys was varied showed strong changes in kinetics during the 180 min reaction course (Supplementary Materials, Figure S2). In the model solutions containing equimolar L-cys and Cu(II), linearity was observed in the ln Cu(I) concentration–time plots during the first minutes of the reaction course (up to 20 min), which is characteristic of first-order reaction kinetics. As the L-cys concentration increased, reaching a tenfold excess over the initial Cu(II) concentration, the linearity disappeared even in the early stages of the reaction course, and the reaction course showed a pattern that was independent of the Cu(I) concentration. A comparison of the calculated rate constants (k, Table S1 in the Supplementary Materials) for ln Cu(I) concentration–time indicates faster Cu(I) oxidation in the presence of L-cys compared to GSH (under the conditions of an equimolar Cu:thiol ratio and under the conditions of linearity in ln Cu(I) concentration–time). The changes in the reaction kinetics over time in the solution with equimolar L-cys and Cu can be attributed to the Fenton-like process. The same reaction pattern was previously observed by Moffet and Zika for Cu(I) oxidation in solutions containing NaCl [55]. The pattern is explained by the existence of a reverse reaction in which formed Cu(II) is reduced with ROS. It is known that the absence of free thiol in the solution produces H_2_O_2_, and Fenton-like reactions between Cu(I)–thiol and ROS occur [54]. During the reverse reaction, Cu(I) was formed and additionally influenced the reaction kinetics, as shown by a change in the reaction slope (k) as well as the reaction order. When L-cys was present in excess relative to the added Cu, the reaction kinetics were independent of the Cu(I) concentration alone, indicating a complex reaction scheme involving oxidation of the free L-cys and the Cu(I)–cys complex, followed by a competition between cystine and L-cys for Cu(I) [60,62,63].

### 2.3. Interaction of Cu and 3-MPA

**Figure 5:**
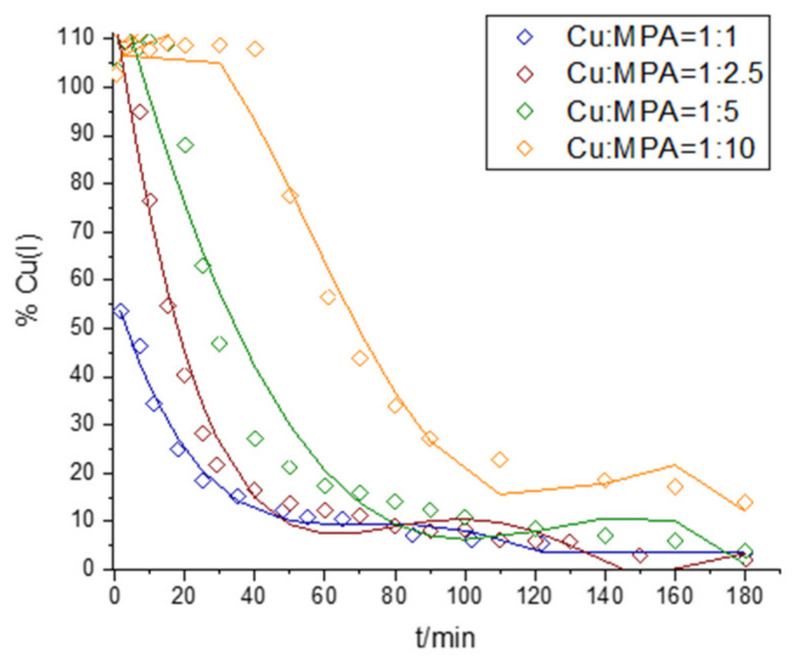
Kinetics of Cu(II) reduction and Cu(I) oxidation in solutions containing 100 µM Cu(II) and 100 µM MPA (blue line), 250 µM MPA (wine red line), 500 µM MPA (green line), or 1000 µM MPA (orange line).

**Figure 6:**
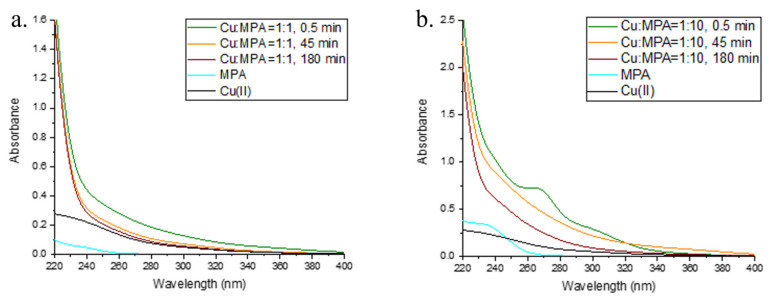
UV–Vis spectra of (**a**) Cu:MPA = 1:1 at t = 0.5 min (green line), t = 45 min (orange line), and t = 180 min (red line). The UV–Vis spectra of a solution containing 100 µM MPA without Cu (cyan line) and 100 µM Cu without MPA (black line) are also shown. (**b**) Cu:MPA = 1:10 at t = 0.5 min (green line), t = 45 min (orange line), and t = 180 min (red line). The UV–Vis spectra of the solution containing 1000 µM MPA without Cu (cyan line) and 100 µM Cu without MPA (black line) are also shown.

The kinetics of Cu(II) reduction and Cu(I) oxidation in solutions containing Cu and MPA in metal:ligand ratios of 1:1, 1:2.5, 1:5, and 1:10 and under oxic conditions at pH = 8.4 are shown in Figure 5 and Figure 6. The complete reduction of Cu(II) took place in all the solutions studied in which MPA was present in excess, while Cu:MPA = 1:1 resulted in a maximum reduction of 52% Cu(II). The maximum reduction occurred within 30 s after adding Cu to a solution containing MPA in 0.1 M borate buffer. In the sample containing Cu:MPA = 1:10, a peak with an A_max_ of 260 nm and a shoulder at 300 nm appeared immediately after the addition of Cu to a solution containing MPA. Similar peaks were observed in Cu:GSH = 1:10 and are addressed as Cu(I)–thiol complexes [52,53]. The peaks disappeared after the start of Cu(I) oxidation, i.e., about 45 min after the start of the reaction course in the mixture with Cu:MPA = 1:10.

Similar to what has already been described in this manuscript, we investigated the reaction kinetics in more detail (Supplementary Materials, Figure S3). The reaction course allowed us to analyze reaction kinetics with respect to the Cu(I) concentration at all four investigated Cu:MPA ratios. An analysis of k for the plots of ln Cu(I)–time (in the parts where linearity was observed) shows the dependence of k on MPA concentration, as shown in Table S1 (Supplementary Materials). The first-order reaction rate (with respect to the Cu(I) concentration) for the Cu(I) oxidation reaction decreased with an increasing MPA concentration. This observed pattern again points to the importance of the experimental conditions used, i.e., the initial ratio of Cu(II) to thiol, as already observed in the case of Cu and GSH and L-cys. When thiol (in this case, MPA) is present in a tenfold excess with respect to the initial concentration of Cu(II), the formation of Cu(I)–MPA is favored and the oxidation of Cu(I) by O_2_ is strongly inhibited as a result of the Cu(I) complexation (Figure 6a). As was already observed (in Section 2.2, where the interaction of Cu with L-cys is investigated), a shift in the reaction kinetics from first to second order occurred over the course of the reaction, which in turn can be explained by the fact that more than just Cu(I) was involved in the reaction mechanism, namely ROS and Cu(II).

### 2.4. Interaction of Cu with TAA 

**Figure 7:**
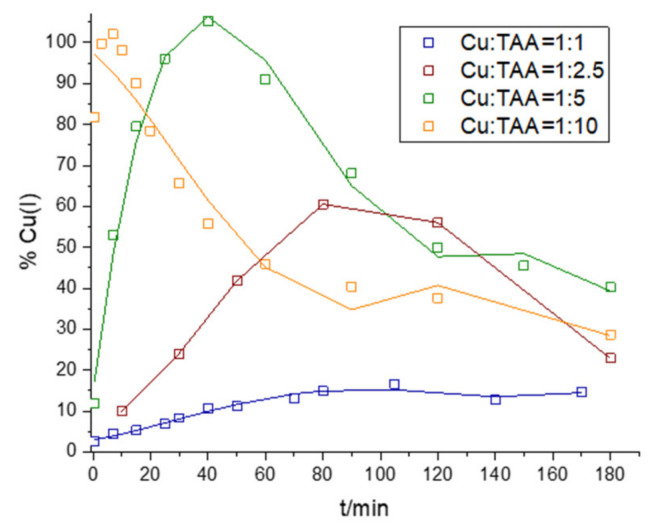
Kinetics of Cu(II) reduction and Cu(I) oxidation in solutions containing 100 µM Cu(II) and 100 µM TAA (blue line), 250 µM TAA (wine red line), 500 µM TAA (green line), or 1000 µM TAA (orange line).

**Figure 8:**
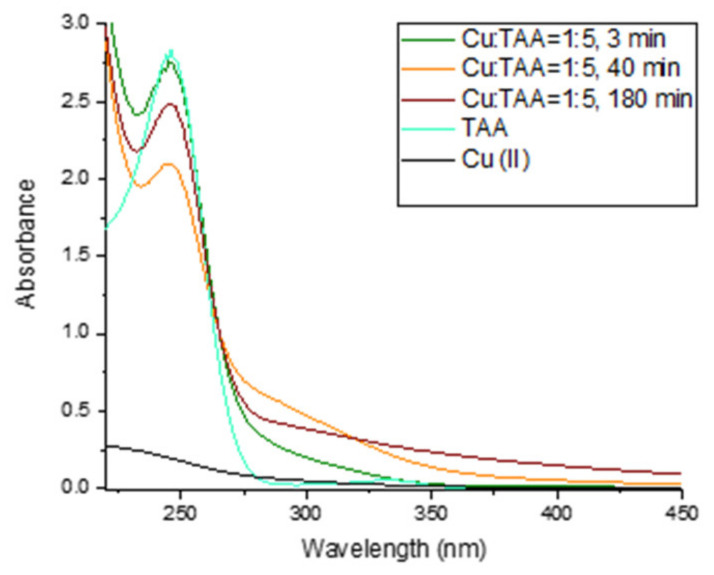
UV–Vis spectra of Cu:TAA = 1:5 at t = 3 min (green line), t = 40 min (orange line), and t = 180 min (red line). The UV–Vis spectra of a solution containing 500 µM TAA without Cu (cyan line) and 100 µM Cu(II) without TAA (black line) are also shown.

The interaction of Cu(II) with TAA was studied by adding an aliquot of Cu(II) to solutions with increasing TAA in 0.1 M borate buffer at pH 8.4, as shown in Figure 7 and Figure 8. Four different ratios of Cu to TAA were studied over 180 min: Cu:TAA = 1:1, Cu:TAA = 1:2.5, Cu:TAA = 1:5, and Cu:TAA = 1:10. Immediately after the addition of Cu(II) to TAA, a reduction of Cu(II) took place, but at a much slower rate than when Cu(II) was mixed with GSH, L-Cys, or MPA. For Cu:TAA = 1:1, the Cu(I) maximum was reached after about 100 min, while for Cu:TAA = 1:2.5, Cu:TAA = 1:5, and Cu:TAA = 1:10, the maximum of Cu(I) was reached after 80, 40, and 7 min, respectively. Both Cu:TAA = 1:5 and Cu:TAA = 1:10 resulted in the 100% reduction of Cu(II), but the maximum was reached faster with a higher Cu:TAA ratio. In addition to monitoring Cu(I) with the BCS assay, UV–Vis spectra of the Cu:TAA = 1:5 solution were recorded because the 1:5 ratio resulted in the 100% reduction of Cu(II), and the reduction was slow enough to monitor with UV–Vis spectrophotometry. The UV–Vis spectrum of TAA in 0.1 M borate buffer without Cu addition resulted in a peak with an A_max_ of 246 nm and a lower peak with an A_max_ of 300 nm. After the addition of Cu(II) to the TAA solution, the peak with an A_max_ of 246 decreased while the Cu(I) concentration increased, indicating a loss of free thiol due to its complexation with Cu(I) and its oxidation after reaction with Cu(II). After 40 min, the concentration of Cu(I) began to decrease (Figure 7), followed by an increase in the peak with an A_max_ of 246 nm (Figure 8), indicating the release of the thiol group bound to Cu. These results are consistent with our recent study on the interaction of vanadates with thiols, which also showed the lower reducing ability of TAA compared to L-cysteine and MPA [64]. It seems very likely that, because of the different structural properties of the thiols studied (different chelating properties, i.e., monodentate vs. bidentate), TAA is not able to stabilize the formed Cu(I) against oxidation and thus exhibits a lower reduction of Cu(II) at lower Cu:TAA ratios. Moreover, the SH group in the TAA molecule is located next to the C=O group, resulting in a resonant structure, which is not the case for any other thiol studied (GSH, L-cys, or MPA), affecting the electron-donating properties of the sulfur atom of TAA [65].

After closely inspecting the reaction kinetics (Supplementary Materials, Figure S4) for Cu:TAA = 1:1, we were able to determine the k value of the reduction reaction of Cu(II) with TAA (Table S1, Supplementary Materials). The figure in the Supplementary Materials (Figure S4a–h) also indicates rapid changes in the reaction sequence as well as in the course of the reaction (reduction vs. oxidation). Unfortunately, we were able to determine k for the Cu(I) oxidation reaction only in the cases where TAA was in five- and tenfold excess with respect to the added Cu (Table S1, Supplementary Materials). However, as noted in the case of L-cys and MPA, the initial reduction of Cu(II) was followed by the Cu(I) oxidation reaction, which, in turn, shows changes in the reaction order from first to second that, again, can be explained as earlier.

## 3. Materials and Methods

### 3.1. Materials

All solutions were prepared with deionized water from the Milli-Q (MQ) system (18.2 MΩ, Millipore, Burlington, MA, USA), and all chemicals were of analytical grade. The Cu(I) standard solution used for the calibration curve of the Cu(I)–bathocuproine complex (see Section 3.2) was prepared by dissolving copper(I) chloride (CuCl; Thermo Fisher Scientific, Waltham, MA, USA) in a solution containing 1 M sodium chloride (NaCl; Grammol, North Salt Lake, UT, USA) and 0.1 M hydrochloric acid (HCl; Roth, Newport Beach, CA, USA), which was previously purged with high purity nitrogen to remove oxygen [66]. The bathocuproine sulfonate disodium salt hydrate (BCS; Thermo Fisher Scientific), was prepared by dissolving BCS in MQ water to a concentration of 1000 µM. The Cu(II) standard solution was prepared by dissolving copper(II) sulfate (CuSO_4_; VWR BDH Prolabo Chemicals, Radnor, PA, USA) in MQ water to a final concentration of 0.01 M Cu(II). Thiol solutions of L-cysteine (L-cys), reduced glutathione (GSH), thioacetic acid (TAA), and 3-mercaptopropionic acid (MPA) were purchased from Thermo Fisher Scientific and prepared fresh daily by dissolving the thiols in MQ water to a final concentration of 0.01 M. A constant ionic strength and a pH = 8.4, relevant to the environmental and physiological conditions, in the model solutions containing Cu and thiol were achieved using 0.1 M borate buffer. The borate buffer was prepared from ortho-boric acid (VWR BDH Prolabo Chemicals), and its pH was adjusted to pH = 8.4 with sodium hydroxide (NaOH; Lach-ner Chemicals, Neratovice, Czech Republic).

Copper reduction by individual thiol species was investigated by measuring the Cu(I) concentration with a UV–Vis spectrophotometer (Analytik Jena, Jena, Germany) in a 1 cm quartz cuvette.

### 3.2. Procedure

The interaction of Cu with thiols was studied by adding aliquots of a Cu(II) stock solution to a solution containing thiol (L-cysteine, glutathione, 3-mercaptopropionic acid, or thioacetic acid) buffered to pH = 8.4 with 0.1 M borate buffer. The Cu concentration of 100 µM was the same in all experiments, while the thiol concentrations ranged from 100 to 1000 µM. The kinetics of the reduction of Cu(II) and the oxidation of Cu(I) were studied by adding aliquots of the Cu–thiol solution to the mixture of BCS and EDTA (BCS assay). Previous studies have shown that BCS effectively binds Cu(I) in an orange complex with an absorption maximum (A_max_) of 484 nm, while a masking ligand is necessary to avoid Cu(II) interference [66,67]. In this study, EDTA was used as the masking ligand for Cu(II) with a fivefold excess of EDTA over BCS, which has been found to be optimal for ensuring Cu(II) complexation while avoiding Cu(I) oxidation [66,67]. A volume of 1 mL of the Cu–thiol model solution was added to the mixture containing 3 mL of BCS (1000 µM) and 0.15 mL of EDTA (0.1 M), resulting in a dilution factor (DF) of 4.15 in the Cu–thiol model solution and final concentrations of 723 µM BCS and 0.00361 M EDTA. The addition of the Cu–thiol solution to the mixture of BCS and EDTA resulted in the formation of an orange-colored solution with an absorption maximum (A_max_) of 484 nm, indicating the presence of Cu(I) in the solution. Cu(I) concentrations were determined by measuring the A_484_ solution, calculating the Cu(I) concentration from the Cu(I)–BCS calibration curve, and correcting the Cu(I) concentration by a dilution factor of 4.15. For the Cu(I)–BCS calibration curve, one blank and five standard additions of Cu(I) were prepared. The blank solution contained 3 mL of 1000 µM BCS, 0.15 mL of 0.1 M EDTA, 0.1 mL of 1 M borate buffer, and MQ water with a final volume of 4.15 mL. The final concentrations of 723 µM BCS and 0.00361 M EDTA were the same as those used to measure the absorbance of Cu–thiol solutions. The Cu(I) standard solutions were prepared in the same way as the blank solution, adding five different Cu(I) aliquots that resulted in a linear absorbance over the concentration range studied, from 1.1 to 35.2 µM. The Cu(I) standard solution was freshly prepared before the experiment, following the procedure described in Section 3.1. To better understand the reaction mechanism, in addition to monitoring the Cu(I) concentration, UV–Vis spectra of solutions containing Cu and L-cys were recorded at the same reaction time as was the Cu(I) determination.

## 4. Conclusions

The study of the interactions of Cu with GSH (glutathione), L-cys (L-cysteine), MPA (3-mercaptopripionic acid), and TAA (thioacetic acid) revealed a complex reaction scheme with a rapid Cu(II) reduction in the case of GSH, L-cys, and MPA. The reduction reaction was rapid (within seconds), and we could instead follow the kinetics of Cu(I) oxidation under conditions where the thiol compound being studied was varied in stoichiometry with respect to the Cu(II) added. An examination of the ln Cu(I) and 1/Cu(I) concentrations over time shows that the reaction kinetics changed over the course of the reaction. Only the solution with Cu:GSH = 1:1 showed first-order reaction kinetics with respect to Cu(I) oxidation, and the kinetics did not change during the 180 min of the reaction course. During the reaction courses of the Cu:L-cys = 1:1 and Cu:MPA = 1:1 mixtures, changes in the reaction kinetics for the Cu(I) oxidation process were observed in the later stages of the reaction course. In the later stages of the reaction, the H_2_O_2_ formed during Cu(I) oxidation with O_2_ accumulated and triggered a Fenton-like reaction. The addition of Cu(I) to the already-ongoing reaction and the involvement of further reactants (Cu(II) and ROS) changed the reaction kinetics, as can be observed. Increasing the thiol concentration also increased the stability of Cu(I) against oxidation, which is probably due to the formation of stable Cu(I) complexes with the investigated thiols. Only in the case of TAA were we able to follow the reduction kinetics of added Cu(II), and the reducing power of the thiols studied can be summarized as follows: L-cys ≅ GSH > MPA > TAA. The lower reducing ability of TAA towards Cu(II) can be explained by its monodentate nature, as well as by the SH group being located next to the C=O group, with respect to the GSH, L-cys, and MPA.

## Data Availability

Data available on request due to restrictions. The data presented in this study are available on request from the corresponding author. The data are not publicly available due to privacy.

## Supplementary Materials

The following supporting information can be downloaded at: https://www.mdpi.com/article/10.3390/molecules28135065/s1, Figure S1: Plots of reaction kinetics for (a) first order reaction, Cu:GSH = 1:1 (b) second order reaction, Cu:GSH = 1:1 (c) first order reaction, Cu:GSH=1:10 (d) second order reaction, Cu:GSH = 1:10; Figure S2: Plots of reaction kinetics for (a) first order reaction, Cu:L-cys = 1:1 (b) second order reaction, Cu:L-cys = 1:1 (c) first order reaction, Cu:L-cys = 1:2.5 (d) second order reaction, Cu:L-cys = 1:2.5 (e) first order reaction, Cu:L-cys = 1:5 (f) second order reaction, Cu:L-cys = 1:5 (g) first order reaction, Cu:L-cys = 1:10 (h) second order reaction, Cu:L-cys = 1:10; Figure S3: Plots of reaction kinetics for (a) first order reaction, Cu:MPA = 1:1 (b) second order reaction, Cu:MPA = 1:1 (c) first order reaction, Cu:MPA = 1:2.5 (d) second order reaction, Cu:MPA = 1:2.5 (e) first order reaction, Cu:MPA = 1:5 (f) second order reaction, Cu:MPA = 1:5 (g) first order reaction, Cu:MPA = 1:10 (h) second order reaction, Cu:MPA = 1:10; Figure S4: Plots of reaction kinetics for (a) first order reaction, Cu:TAA = 1:1 (b) second order reaction, Cu:TAA = 1:1 (c) first order reaction, Cu:TAA = 1:2.5 (d) second order reaction, Cu:TAA = 1:2.5 (e) first order reaction, Cu:TAA = 1:5 (f) second order reaction, Cu:TAA = 1:5 (g) first order reaction, Cu:TAA = 1:10 (h) second order reaction, Cu:TAA = 1:10; Table S1: Reaction rate constants (*k*) for reactions of Cu(II) reduction and Cu(I) oxidation.

## References

[1] Linder M.C., Frieden E. (1991). Introduction and overview of copper as an element essential for life. Biochemistry of Copper.

[2] Rensing C., McDevitt S.F. (2013). The copper metallome in prokaryotic cells. Met. Ions Life. Sci..

[3] Peña M.M., Lee J., Thiele D.J. (1999). A delicate balance: Homeostatic control of copper uptake and distribution. J. Nutr..

[4] Rucker R.B., Kosonen T., Clegg M.S., Mitchell A.E., Uriu-Hare J.Y. (1998). Copper, lysyl oxidase, and extracelullar matrix protein cross linking. Am. J. Clin. Nutr..

[5] Solano F. (2018). On the metal cofactor in the tyrosinase family. Int. J. Mol. Sci..

[6] Raposo B., Rodríguez C., Martínez-González J., Badimon L. (2004). High levels of homocysteine inhibit lysyl oxidase (LOX) and downregulate LOX expression in vascular endothelial cells. Atherosclerosis.

[7] Park Y.D., Lyou Y.J., Hahn H.S., Hahn M.J., Yang J.M. (2006). Complex inhibition of tyrosinase by thiol-composed Cu^2+^ Chelators: A Clue for Designing Whitening Agents. J. Biomol. Struct. Dyn..

[8] Bakavayev S., Chetrit N., Zvagelsky T., Mansour R., Vyazmensky M., Barak Z., Israelson A., Engel S. (2019). Cu/Zn-superoxide dismutase and wild-type like fALS SOD1 mutants produce cytotoxic quantities of H_2_O_2_ via cysteine-dependent redox short-circuit. Sci. Rep..

[9] Klotz L.O., Krö K.D., Buchczyk D.P., Sies H. (2003). Role of copper, zinc, selenium and tellurium in the cellular defense against oxidative and nitrosative stress. J. Nutr..

[10] Bremner I. (1998). Manifestations of Cu excess. Am. J. Clin. Nutr..

[11] Hodgson E.K., Fridovich I. (1975). The interaction of bovine erythrocyte superoxyde dismutase with hydrogen peroxide: Inactivation of the enzyme. Biochemistry.

[12] Winterbourn C.C., Peskin A.V., Parsons-Mair H.N. (2002). Thiol oxidase activity of copper, zinc superoxide dismutase. J. Biol. Chem..

[13] Gaetke L.M., Chow-Johnson H.S., Chow C.K. (2014). Copper: Toxicological relevance and mechanisms. Arch. Toxicol..

[14] Gaetke L.M., Chow C.K. (2003). Copper toxicity, oxidative stress, and antioxidant nutrient. Toxicology.

[15] Kumar P., Tewari R.K., Sharma P.N. (2008). Modulation of copper toxicity-induced oxidative damage by excess supply of iron in maize plants. Plant. Cell. Rep..

[16] Valko M., Morris H., Cronin M.T.D. (2005). Metals, toxicity and oxidative stress. Curr. Med. Chem..

[17] Murakami K., Tsubouchi R., Fukayama M., Yoshino M. (2014). Copper-dependent inhibition and oxidative inactivation with affinity cleavage of yeast glutathione reductase. Biometals.

[18] Xiao Y., Chen D., Zhang X., Cui X., Fan J., Bi C., Dou Q.P. (2010). Molecular study on copper-mediated tumor proteasome inhibition and cell death. Int. J. Oncol..

[19] Prohaska J.R. (2008). Role of copper transporters in copper homeostasis. Am. J. Clin. Nutr..

[20] Shenberger Y., Marciano O., Gottlieb H.E., Ruthstein S. (2018). Insights into the N-terminal Cu(II) and Cu(I) binding sites of the human copper transporter CTR1. J. Coord. Chem..

[21] Rubino J.T., Chenkin M.P., Keller M., Riggs-Gelasco P., Franz K.J. (2011). A comparison of methionine, histidine and cysteine in copper(I)-binding peptides reveals differences relevant to copper uptake by organisms in diverse environments. Metallomics.

[22] Hatori Y., Lutsenko S. (2013). An expanding range of functions for the copper chaperone/antioxidant protein atox1. Antioxid. Redox Signal..

[23] Hatori Y., Clasen S., Hasan N.M., Barry A.N., Lutsenko S. (2012). Functional partnership of the copper export machinery and glutathione balance in human cells. J. Biol. Chem..

[24] Heaton D.N., George G.N., Garrison G., Winge D.R. (2001). The mitochondrial copper metallochaperone Cox17 exists as an oligomeric, polycopper complex. Biochemistry.

[25] Banci L., Bertini I., Ciofi-Baffoni S., Hadjiloi T., Martinelli M., Palumaa P. (2008). Mitochondrial copper(I) transfer from cox17 to sco1 is coupled to electron transfer. Proc. Natl. Acad. Sci. USA.

[26] Moffet J.W., Zika R.G., Brand L.E. (1990). Distribution and potential sources and sinks of copper chelators in the Sargasso sea. Deep Sea Res..

[27] Moffett J.W., Brand L.E. (1996). Production of strong, extracellular Cu chelators by marine cyanobacteria in response to Cu stress. Limnol. Oceanogr..

[28] Whitby H., Posacka A.M., Maldonado M.T., van den Berg C.M.G. (2018). Copper-binding ligands in the NE Pacific. Mar. Chem..

[29] Tang D., Shafer M.M., Karner D.A., Armstrong D.E. (2005). Response of nonprotein thiols to copper stress and extracellular release of glutathione in the diatom Thalassiosira Weissflogii. Limnol. Oceanogr..

[30] Valent I., Bednárová L., Schreiber I., Bujdák J., Valachová K., Šoltés L. (2022). Reaction of N-Acetylcysteine with Cu^2+^: Appearance of intermediates with high free radical scavenging activity: Implications for anti-/pro-oxidant properties of thiols. Int. J. Mol. Sci..

[31] Smith R.C., Reed V.D., Hill W.E. (1994). Oxidation of thiols by copper(II). Phosphorus Sulfur Silicon Relat. Elem..

[32] Anderson M.E. (1998). Glutathione: An overview of biosynthesis and modulation. Chem. Biol. Interact..

[33] Freedman J.H., Ciriolo M.R., Peisach J. (1989). The role of glutathione in copper metabolism and toxicity. J. Biol. Chem..

[34] Maryon E.B., Molloy S.A., Kaplan J.H. (2013). Cellular glutathione plays a key role in copper uptake mediated by human copper transporter 1. Am. J. Physiol..

[35] Hasanuzzaman M., Nahar K., Anee T.I., Fujita M. (2017). Glutathione in plants: Biosynthesis and physiological role in environmental stress tolerance. Physiol. Mol. Biol. Plants.

[36] Dupont C.L., Ahner B.A. (2005). Effects of copper, cadmium, zinc on the production and exudation of thiols by Emiliania Huxleyi. Limnol. Oceanogr..

[37] Leal M.F.C., Vasconcelos T.D.M.S., van den Berg C.M.G. (1999). Copper-induced release of complexing ligands similar to thiols by Emiliania Huxleyi in seawater cultures. Limnol. Oceanogr..

[38] Hu H., Mylon S.E., Benoit G. (2006). Distribution of the thiols glutathione and 3-mercaptopropionic acid in Connecticut Lakes. Limnol. Oceanogr..

[39] Tang D., Shafer M., Karner D.A., Overdier J., Armstrong D.E. (2004). Factors affecting the presence of dissolved glutathione in estuarine waters. Environ. Sci. Technol..

[40] Liem-Nguyen V., Bouchet S., Björn E. (2015). Determination of sub-nanomolar levels of low molecular mass thiols in natural waters by liquid chromatography tandem mass spectrometry after derivatization with p-(hydroxymercuri) benzoate and online preconcentration. Anal. Chem..

[41] Swarr G.J., Kading T., Lamborg C.H., Hammerschmidt C.R., Bowman K.L. (2016). Dissolved low-molecular weight thiol concentrations from the U.S. GEOTRACES North Atlantic Ocean zonal transect. Deep Sea Res..

[42] Van Den Berg C.M.G., Househam B.C., Riley J.P. (1988). Determination of cystine and cysteine in seawater using cathodic stripping voltammetry in the presence of Cu(II). J. Electroanal. Chem. Interfacial Electrochem..

[43] Takagi H., Ohtsu I., Yokota A., Ikeda M. (2017). L-cysteine metabolism and fermentation in microorganisms. Advances in Biochemical Engineering/Biotechnology.

[44] Poole L.B. (2015). The basics of thiols and cysteines in redox biology and chemistry. Free Radic. Biol. Med..

[45] Pushie M.J., Zhang L., Pickering I.J., George G.N. (2012). The fictile coordination chemistry of cuprous-thiolate sites in copper chaperones. Biochim. Biophys. Acta Bioenerg..

[46] Kiene R.P., Taylor B.F. (1988). Biotransformations of organosulphur compounds in sediments via 3-mercaptopropionate. Nature.

[47] Allen K.D., White R.H. (2016). Occurrence and biosynthesis of 3-mercaptopropionic acid in Methanocaldococcus Jannaschii. FEMS Microbiol. Lett..

[48] Hill J. (2000). Sulfur and the Origins of Life. Master’s Thesis.

[49] Chandru K., Gilbert A., Butch C., Aono M., Cleaves H.J. (2016). The abiotic chemistry of thiolated acetate derivatives and the origin of life. Sci. Rep..

[50] Sanden S.A., Yi R., Hara M., McGlynn S.E. (2020). Simultaneous synthesis of thioesters and iron-sulfur clusters in water: Two universal components of energy metabolism. Chem. Comm..

[51] Battin E.E., Brumaghim J.L. (2008). Metal specificity in DNA damage prevention by sulfur antioxidants. J. Inorg. Biochem..

[52] Yin S.N., Liu Y., Zhou C., Yang S. (2017). Glutathione-mediated Cu(I)/Cu(II) Complexes: Valence-dependent effects on clearance and in vivo imaging application. Nanomaterials.

[53] Aliaga M.E., López-Alarcón C., Bridi R., Speisky H. (2016). Redox-implications associated with the formation of complexes between copper ions and reduced or oxidized glutathione. J. Inorg. Biochem..

[54] Kachur A.V., Koch C.J., Biaglow J.E. (1998). Mechanism of copper-catalyzed oxidation of glutathione. Free Radic. Res..

[55] Moffet J.W., Zika R.G. (1983). Oxidation kinetics of Cu(I) in seawater: Implications for its existance in the marine environment. Mar. Chem..

[56] Ngamchuea K., Batchelor-Mcauley C., Compton R.G. (2016). The copper(II)-catalyzed oxidation of glutathione. Eur. J. Chem..

[57] Corazza A., Harvey I., Sadler P.J. (1996). LH,13C-NMR and X-Ray absorption studies of copper(I) glutathione complexes. Eur. J. Biochem..

[58] Aliaga M.E., Carrasco-Pozo C., López-Alarcón C., Speisky H. (2010). The Cu(I)-glutathione complex: Factors affecting its formation and capacity to generate reactive oxygen species. Trans. Met. Chem..

[59] Pecci L., Montefoschi G., Musci G., Cavallini D. (1997). Novel findings on the copper catalysed oxidation of cysteine. J. Amino Acids.

[60] Rigo A., Corazza A., Luisa Di Paolo M., Rossetto M., Ugolini R., Scarpa M. (2004). Interaction of copper with cysteine: Stability of cuprous complexes and catalytic role of cupric ions in anaerobic thiol oxidation. J. Inorg. Biochem..

[61] Cavallini D., De Marco C., Dupre S., Rotilio G. (1969). The copper catalyzed oxidation of cysteine to cystine. Arch. Biochem. Biophys..

[62] Kolthoff I.M., Stricks W. (1951). Polarographic investigations of reactions in aqueous solutions containing copper and cysteine (cystine). J. Am. Chem. Soc..

[63] Flitman R., Frieden E. (1957). Cuprous ion formation in cupric ion catalyzed oxidations. J. Am. Chem. Soc..

[64] Knežević L., Bura-Nakić E. (2023). Investigation of thiol compounds (L-cysteine, thioacetic acid and ethanethiol) with V(V) and V(IV) using combined spectroscopy and chromatography. J. Inorg. Biochem..

[65] Fausto R., Batista de Carvalho L.A.E., Teixeira-Dias J.J.C. (1990). Molecular structure and properties of thioacetic acid. J. Mol. Struct..

[66] Moffet J.W., Zika R.G., Petasne R.G. (1985). Evaluation of bathocuproine for the spectrophotometric determination of copper (I) in copper redox studies with applications in studies of natural waters. Anal. Chim. Acta.

[67] Buerge-Weirich D., Sulzberger B. (2004). Formation of Cu(I) in estuarine and marine waters: Application of a new solid-phase extraction method to measure Cu(I). Environ. Sci. Technol..

