# Supplementary material for: Interaction between Cu and Thiols of Biological and Environmental Importance: Case Study Using Combined Spectrophotometric/Bathocuproine Sulfonate Disodium Salt Hydrate (BCS) Assay
Source: Molecules. 2023 Jun 28;28(13):5065. doi: 10.3390/molecules28135065 (PMC10343574; doi:10.3390/molecules28135065)
Supplement: Supplementary file 1 [file molecules-28-05065-s001.zip › molecules-2451738-supplementary.pdf]

Figure S1 shows plots of ln absorbance (lnA) for Cu (I) as a function of time (Figure S1a,c), representative of the first order reaction, and plots for 1/A as a function of time, representative of the second order reaction (S1b, S1d), for solutions containing Cu and GSH in different ratios. For Cu:GSH=1:1, linearity of lnA with respect to time is observed for most of the reaction course, indicating a first-order reaction (Figure S1a). In the case of GSH excess with respect to the initial Cu(II) concentration, the Cu(I) concentration was constant for the first 60 min of the reaction course, when it was replaced by rapid Cu(I) oxidation. It can be seen from the diagrams in Figure 1c,d that the reaction kinetics within the first 60 minutes do not follow either first or second order reaction kinetics. The black line (Figure S1a) represents the linearity of lnA versus time used to calculate the reaction rate constant (*k*) which is displayed in Table S1 (Supplementary Material).

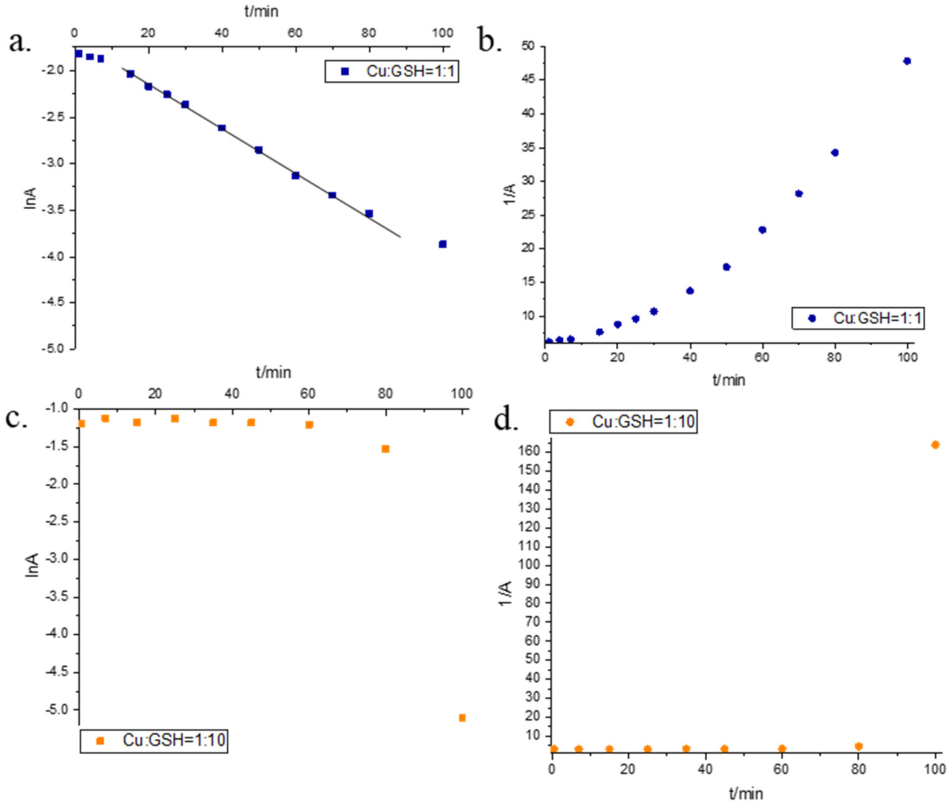

Figure S1. Plots of reaction kinetics for (a) first order reaction, Cu:GSH=1:1 (b) second order reaction, Cu:GSH=1:1 (c) first order reaction, Cu:GSH=1:10 (d) second order reaction, Cu:GSH=1:10.

Figure S2 shows plots of ln absorption (lnA) for Cu (I) as a function of time (Figures S2a, S2c, S2e, S2g) representative of the first order reaction and plots for 1/A as a function of time representative of the second order reaction (Figures S2b, S2d, S2f, S2h) for solutions containing Cu and L-cys in different ratios. For Cu:L-cys=1:1, a linearity of lnA is observed compared to the time at the beginning of the reaction, which is characteristic of the first order reaction (Figure S2a). However, the second reaction phase is characterized by a linearity of 1/A with respect to time, implying a shift from the first order reaction to the second order reaction in the later reaction phase (Figure S2b). In the case of the L-cys excess with respect to the initial Cu concentration, a non-linearity of both lnA with respect to time and 1/A with respect to time is observed, indicating that the reaction kinetics change over the time of the reaction course (Figure S2c-S2f). The black lines represent the linearity of lnA versus time and 1/A versus A, where the linearity of lnA versus time is used to calculate the reaction rate constant (*k*).

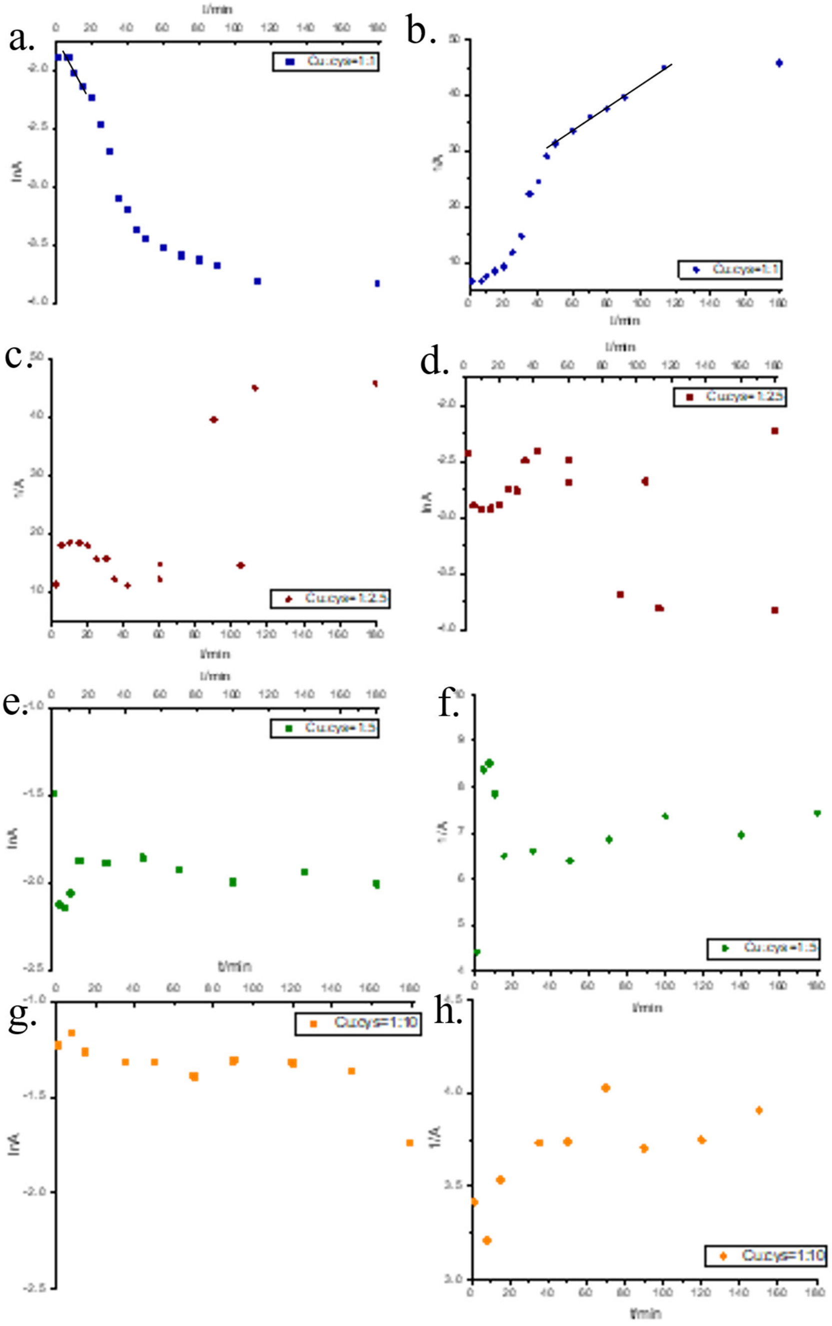

Figure S2. Plots of reaction kinetics for a.) first order reaction, Cu:Lcys=1:1 b.)second order reaction, Cu:L-cys=1:1 c.) first order reaction, Cu:L-cys=1:2.5 d.) second order reaction, Cu:L-cys=1:2.5 e.) first order reaction, Cu:L-cys=1:5 f.) second order reaction, Cu:L-cys=1:5 g.) first order reaction, Cu:L-cys=1:10 h.) second order reaction, Cu:L-cys=1:10.

Figure S3 shows plots of ln absorption (lnA) for Cu (I) as a function of time (Figures S3a, S3c, S3e, S3g), representative of the first order reaction, and plots for 1/A as a function of time, representative of the second order reaction (Figures S3b, S3d, S3f, S3h), for solutions containing Cu and MPA in different ratios. For Cu:MPA=1:1, the linearity of lnA is not observed (Figure S3a). Instead, linearity between 1/A and time is observed, indicating a second order reaction at the beginning of the reaction course (Figure S3b). In the case of the MPA excess with respect to the initial Cu concentration, linearity versus time is observed for both lnA and 1/A, indicating that the reaction kinetics change over time of the reaction course (Figure S3c-S3f). The black lines represent the linearity of lnA versus time and 1/A versus A, where the linearity of lnA versus time is used to calculate the reaction rate constant (k).

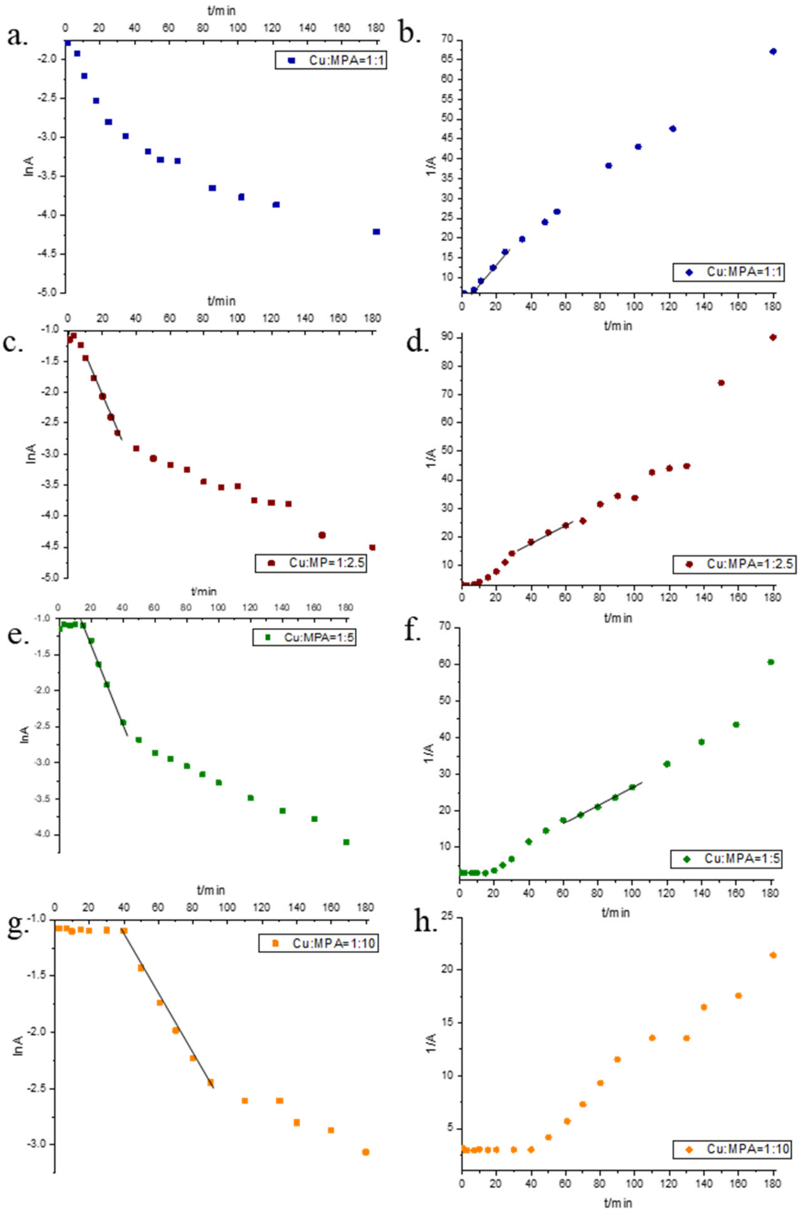

Figure S3. Plots of reaction kinetics for a.) first order reaction, Cu:MPA=1:1 b.) second order reaction, Cu:MPA=1:1 c.) first order reaction, Cu:MPA=1:2.5 d.) second order reaction, Cu:MPA=1:2.5 e.) first order reaction, Cu:MPA=1:5 f.) second order reaction, Cu:MPA=1:5 g.) first order reaction, Cu:MPA=1:10 h.) second order reaction, Cu:MPA=1:10.

Figure S4 shows plots of ln absorbance (lnA) for Cu (I) as a function of time (Figures S4a, S4c, S4e, S4g), representative of the first-order reaction, and plots of 1/A as a function of time, representative of the second-order reaction (Figures S4b, S4d, S4f, S4h), for solutions containing Cu and TAA in different ratios. In contrast to the results in Figures S1, S2 and S3 in the Supplementary Material of the manuscript, the nature of the interaction between Cu and TAA allows us to follow the reduction rates in the early stages of the reaction (up to 80 minutes). For Cu: TAA =1:1, the linearity of lnA is observed as a function of time in the first 40 minutes of the reaction course (Figure S4a), which allows the determination of *k* for the reduction reaction (Table S1, Supplementary Material). Due to the rapid changes in the reaction order as well as in the reaction course (reduction vs. oxidation), we were able to determine *k* for the Cu(I) oxidation reaction for Cu: TAA =1:5 and Cu: TAA =1:10 (Table S1, Supplementary Material). The black lines represent the linearity of lnA versus time and 1/A versus A, where the linearity of lnA versus time is used to calculate the reaction rate constant (*k*).

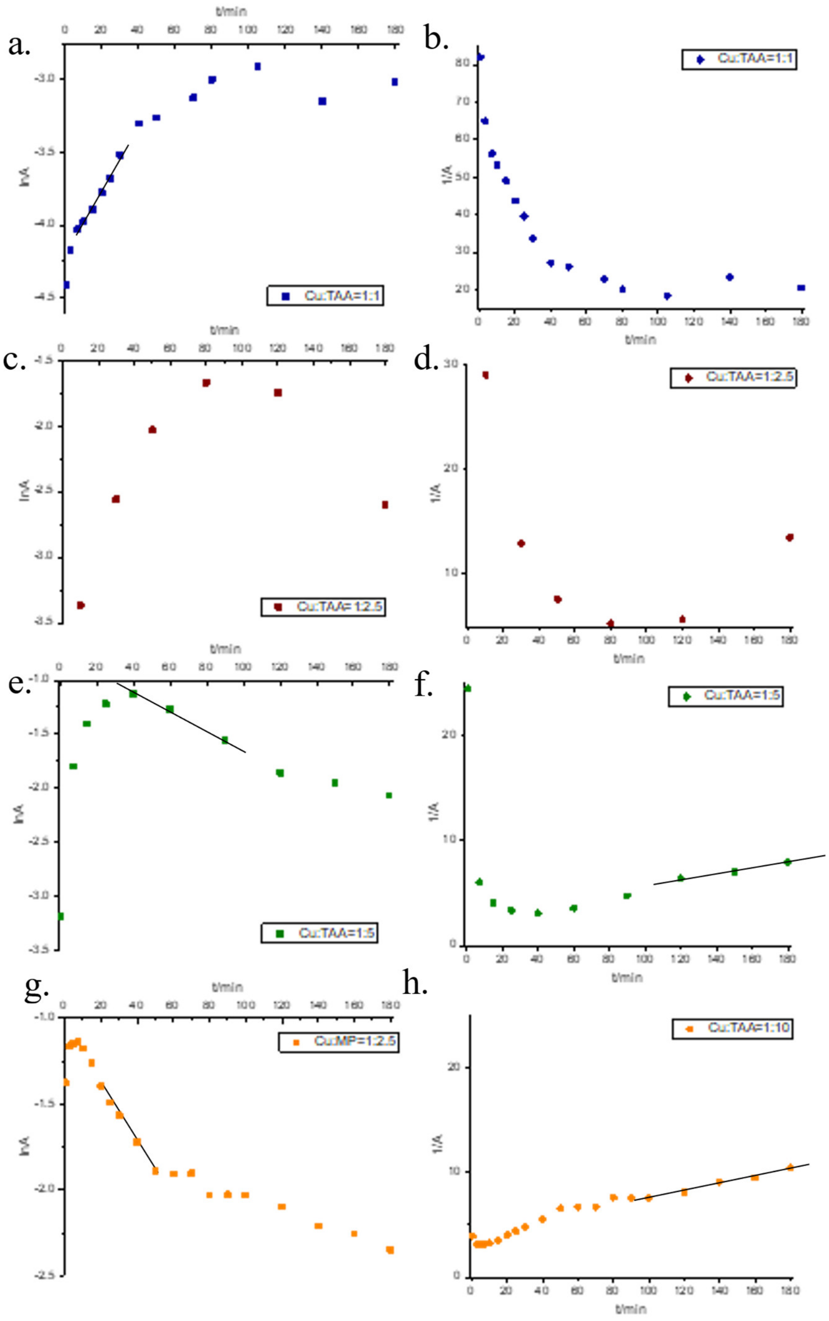

Figure S4. Plots of reaction kinetics for a.) first order reaction, Cu:TAA=1:1 b.) second order reaction, Cu:TAA=1:1 c.) first order reaction, Cu:TAA=1:2.5 d.) second order reaction, Cu:TAA=1:2.5 e.) first order reaction, Cu:TAA=1:5 f.) second order reaction, Cu:TAA=1:5 g.) first order reaction, Cu:TAA=1:10 h.) second order reaction, Cu:TAA=1:10.

Table S1. Reaction rate constants (*k*) for reactions of Cu(II) reduction and Cu(I) oxidation.

Thiol	Reaction	Cu:thiol ratio	k/min^{-1}
L-cys	Cu(I) oxidation	1:1	0.0040
GSH	Cu(I) oxidation	1:1	0.0024
MPA	Cu(I) oxidation	1:2.5	0.0646
	Cu(I) oxidation	1:5	0.0551
	Cu(I) oxidation	1:10	0.0269
TAA	Cu(II) reduction	1:1	0.0196
	Cu(I) oxidation	1:5	0.0098
	Cu(I) oxidation	1:10	0.0161
